# Daily Bi-directional effects of women’s social media-based appearance comparisons, body satisfaction, and disordered eating urges

**DOI:** 10.1186/s40337-024-01096-8

**Published:** 2024-09-03

**Authors:** Jade Portingale, Simone Girardin, Shanshan Liu, Matthew Fuller-Tyszkiewicz, Isabel Krug

**Affiliations:** 1 Melbourne School of Psychological Sciences, The University of Melbourne, North Melbourne VIC 3051, Melbourne, VIC Australia; 2 School of Psychology, Deakin University, Geelong, VIC Australia; 3 Centre for Social and Early Emotional Development, Deakin University, Burwood, VIC Australia

**Keywords:** Appearance comparison, Social media, Body satisfaction, Disordered eating, ecological momentary assessment, Daily monitoring

## Abstract

We assessed the bi-directional relationships between upward appearance comparisons on social media with body dissatisfaction (BD) and disordered eating (DE) in women’s daily lives and the potential moderating effect of trait self-objectification on these relationships. Women (*N* = 315) completed a baseline measure of trait self-objectification, then reported momentary experiences of social media-based appearance comparisons (upward, lateral, downward), body satisfaction, and DE urges (restrict food intake, exercise, overeat) for seven days. We hypothesized that upward (relative to no) comparisons would predict lower body satisfaction and higher DE urges, while lateral and downward (relative to no) comparisons would predict higher body satisfaction and lower DE urges. We expected these relationships to be bi-directional and moderated by trait self-objectification. Multilevel modelling results revealed complex bi-directional relationships. Upward comparisons predicted lower body satisfaction and increased urges to restrict food intake, which in turn predicted increased upward comparisons. Unexpectedly, urges to restrict food intake predicted all comparison types. We observed somewhat unanticipated bi-directional relationships between lateral comparisons and exercise urges, and between downward comparisons and body satisfaction. Uni-directional relationships emerged between upward comparisons and the urge to overeat. Trait self-objectification moderated very few of these relationships. These findings support the non-uniform impact of appearance comparisons on body image and eating concerns and highlight the complexity of daily social media-body image dynamics. Future research using refined measures over extended periods is needed to elucidate these relationships further and inform targeted interventions.

**Supplementary Information:**

The online version contains supplementary material available at 10.1186/s40337-024-01096-8.

## Abstract (plain language summary)

This study examined how comparing oneself to others on social media relates to body satisfaction and disordered eating urges in women’s daily lives, and whether self-objectification influences these relationships. For seven days, 315 women reported their social media appearance comparisons, body satisfaction, and urges related to eating and exercise. Results revealed complex, bi-directional relationships. Upward comparisons (viewing others as more attractive) predicted lower body satisfaction and increased urges to restrict food intake, which in turn led to more upward comparisons. Unexpectedly, urges to restrict food intake predicted all types of comparisons. The study found some surprising bi-directional relationships between lateral comparisons and exercise urges, and between downward comparisons and body satisfaction. Self-objectification had limited moderating effects. These findings highlight the intricate dynamics between social media use and body image concerns, emphasizing the need for further research to inform targeted interventions.

**Supplementary Information:**

The online version contains supplementary material available at 10.1186/s40337-024-01096-8.

## Introduction

As of 2023, 80.4% of Australians were active social media users, with adults spending approximately 2.5 h on social media per day [1]. However, growing research has implicated social media use—specifically, certain forms of engagement and content exposure—in women’s body dissatisfaction (BD) and disordered eating (DE) [2, 3]. Online social appearance comparison is argued to be a central mechanism underlying the detrimental impact of women’s social media use on body image and eating concerns [2, 4]. Research, although scant, has also suggested that high (relative to low) levels of body image concerns and DE symptoms may increase engagement in appearance comparison [5].

According to social comparison theory [6] individuals have an intrinsic desire to evaluate their social standing in various aspects of life and, without objective standards, compare themselves to others. Central to this process is the selection of the comparison target: upward, lateral, or downward which refers to comparisons to other’s self-perceived as better, similar, or less than oneself, respectively (e.g., in terms of physical attractiveness or body weight/shape). Upward comparisons are believed to result in negative body image (e.g., lowered self-perceived attractiveness), whilst downward or lateral comparisons are assumed to improve body image by relieving anxiety about appearance (e.g., weight). Accordingly, research has shown that upward appearance comparisons are associated with body image and eating pathology among women; however, most of these studies were cross-sectional (e.g., [7]) and very few directly contrasted the effects of different directions of comparisons, with some producing mixed findings [8]. Moreover, lateral appearance comparisons remain less examined in this realm (e.g. [5]). Further research is needed to differentiate the direction of appearance comparison in conjunction with other factors, such as the medium of comparison (e.g., online, in-person).

Evidence supports the adverse effect of traditional media (e.g., television, magazines) on women’s body image concerns [8], though this effect may be amplified by social media use. Unlike traditional media, social media offers women the opportunity to compare their appearance with various known targets (e.g., friends, peers, and family), not just celebrities and strangers [9]. Research has demonstrated that appearance comparisons to peers are more strongly associated with young women’s body image concerns, presumably because their attractive appearance is perceived to be more personally attainable (and thus, worthy of pursuit) than that of celebrities [7]. Social media also invites users to create their own content [10] which could facilitate and enhance the desire to obtain appearance ideals. Moreover, unlike traditional media, social media makes users both constant sources and recipients of appearance-related feedback (e.g., via likes and comments) which may encourage greater appearance comparisons [10, 11].

### Social media-based appearance comparisons influencing BD and DE

Experimental studies have increasingly shown that frequent upward appearance comparisons on social media led to immediate increases in negative mood and BD (e.g., [12, 13]). However, these studies often lack ecological validity. A handful of studies have explored whether these lab-based effects generalise to patterns of behaviour in daily life using ecological momentary assessment (EMA) methodology. EMA involves collecting self-report data on current affective and cognitive states and recent behaviours. These studies demonstrated that women were more likely to make upward (relative to downward or lateral) comparisons and these tended to have the most adverse impact on BD and DE outcomes (e.g., [11, 14–17]).

To date, only one EMA study by Fardouly et al. [11] has assessed whether and how the context of appearance comparison (social media, magazines, in-person) affected appearance satisfaction, mood, and diet and exercise thoughts and behaviours. This study (*N* = 146) demonstrated that undergraduate women engaged in more appearance comparison through social media than traditional media and that upward appearance comparisons through social media were associated with more appearance dissatisfaction and thoughts about dieting and exercise (than in-person comparisons) and more negative mood (than comparisons in any other context). This small body of research suggests that upward social media-based appearance comparisons may be particularly harmful to young women’s physical and mental health.

### BD and DE influencing social media-based appearance comparisons

Theoretically, momentary experiences of BD and DE may foster or heighten one’s desire to compare themselves against the standard they aspire to or display selective attention towards individuals they perceive as more attractive due to an underlying tendency to engage in behaviours that confirm their dysfunctional, negative body-related schema. However, the effects of BD and DE on appearance comparison have been comparatively less examined. Nonetheless, in a review of EMA-based studies, Fuller-Tyszkiewicz [5] noted that individuals with elevated DE pathology and/or body dissatisfaction are more likely to report more frequent upward appearance comparisons. These findings suggest a bi-directional relationship between BD/DE and upward appearance comparisons that may reinforce these negative experiences [5].

### Moderating effect of self-objectification

Although informative, social comparison theory [6] does not explain why certain women are particularly susceptible to increases in BD and DE after engaging in appearance comparisons [18]. Fredrickson and Roberts’ [19] objectification theory offers a complementary perspective for elucidating this individual variability. The theory proposes that women in cultures that sexualize the female body are socialized to internalize an observer’s perspective, self-objectifying by habitually monitoring and evaluating their appearance against idealized media standards.

Strelan and Hargreaves’ [20] ‘circle of objectification’ theory suggests a bidirectional, cyclical relationship between this self-objectification and negative psychological consequences like BD and DE. Empirically, self-objectification has been linked to heightened BD [20] and DE symptoms [21], moderating relationships between specific appearance-focused behaviours like posting selfies and eating disorder symptoms [22].

Integrating social comparison and objectification theories may increase understanding of processes underlying appearance-related body image disturbances in women [23]. Theoretically, trait-level (i.e., stable) self-objectification may moderate (strengthen) the relationship between appearance comparisons and body image/eating concerns (and vice versa). Specifically, those high in self-objectification may place greater importance on appearance, potentially amplifying the negative impact of upward comparisons relative to those low in self-objectification. Empirically, Yang et al. [24] found that self-objectification moderated (strengthened) the indirect relationship between selfie viewing and BD via appearance comparisons.

### The current study

The present study was the first to use EMA to examine the bi-directional relationships between social media-based appearance comparisons (upward, lateral, downward) with body satisfaction and DE throughout daily life in a community-based sample of women. We also explored a premise of the ‘circle of objectification’ [20] theory by examining the novel moderating effect of self-objectification on these relationships. Given our community-based sample, urges to engage in DE were measured as a substitute for actual engagement, as the former has been shown to precede the latter and may shed light on the aetiology of eating disorders [25]. We separated DE into the urge to restrict food intake, exercise, and overeat which are common DE practices used to address BD and control body weight/shape among women from the general community (e.g., [14]).

We hypothesized that engaging in upward social media-based appearance comparisons (relative to no comparison) would predict lower levels of body satisfaction and higher levels of DE urges at the state level, while lateral or downward comparisons (relative to no comparison) would have opposite effects on body satisfaction and DE urges (H1). We expected these state-level relationships to be bidirectional (H2). Additionally, we explored the potential moderating role of trait-based self-objectification in these relationships. While we anticipated that self-objectification would amplify the effects of upward comparisons, its impact on lateral and downward comparisons was less clear. Therefore, we examined whether trait-level self-objectification would moderate the associations between all types of appearance comparisons and body satisfaction/DE urges (H3a), as well as their reciprocal influences (H3b), without specifying directional hypotheses for lateral and downward comparisons.

## Method

### Participants

Following ethical approval from a university in Melbourne, we recruited participants from the Research Experience Program (REP) at the university and various sources within the broader community via online advertisements, including social media posts. From 2019 to 2022, 779 women signed up for the study, of which 488 completed both baseline and EMA surveys; however, 173 were excluded as they completed less than 50% of the EMA surveys. This approach was employed to reduce potentially biased results due to missing data (Shiffman et al., 2008) by preserving a spacing of 1–2 h between assessment intervals. This ensured that the participants retained in the sample completed a comparable number of surveys to related studies that have observed effects between EMA-assessed variables within the body image and eating disorder literature [5]. Lastly, 58 participants were excluded as they did not engage in appearance comparisons during the EMA phase.

As displayed in Table 1, retained participants and those excluded due to low compliance did not significantly differ in sociodemographic variables apart from age, the highest level of education completed, and sexual orientation. Retained participants were more likely to be younger and pansexual, whilst excluded participants were more likely to sexually identify as ‘other’ and have a Bachelor’s degree. The final sample comprised 315 participants. On average, women were 20 years old with a body mass index (BMI) of 21.9. Most participants were Caucasian or Asian, spoke English as their primary language, were heterosexual, single, and had completed year 12 or below. Moreover, 30% of participants were classified as at-risk for an eating disorder according to the Eating Attitude Test-26 (EAT-26; [26]) and 8% reported a lifetime eating disorder diagnosis.

**Table 1:** Compliance and demographic comparison between dropped and retained participants

	Excluded (*N* = 173)	Retained (*N* = 315)	Overall (*N* = 488)	*p*^
Compliance (*M*; *SD*)	19.0 (12.8)	33.4 (5.85)	28.3 (11.3)	
Age (*M*; *SD*)	20.8 (4.67)	20.0 (3.87)	20.3 (4.19)	**0.036**
BMI (kg/m^2^; *M*; *SD*)	21.5 (3.63)	21.9 (4.05)	21.8 (3.91)	0.200
Ethnicity (*n*; %)				
Caucasian	61 (35.3%)	136 (43.2%)	197 (40.4%)	0.488
Eastern Asian	41 (23.7%)	70 (22.2%)	111 (22.7%)	
Southern /Southeast Asian	48 (27.7%)	71 (22.5%)	119 (24.4%)	
Middle Eastern	3 (1.7%)	7 (2.2%)	10 (2.0%)	
Other	20 (11.6%)	31 (9.8%)	51 (10.5%)	
Highest level of education completed (*n*; %)				**0.038**
Year 12 or below	119 (68.8%)	249 (79.0%)	368 (75.4%)	
Bachelor’s degree	35 (20.2%)	35 (11.1%)	70 (14.3%)	
Certificate/diploma	9 (5.2%)	17 (5.4%)	26 (5.3%)	
Postgraduate degree	10 (5.8%)	14 (4.4%)	24 (4.9%)	
Main language (*n*; %)				0.196
English	105 (60.7%)	211 (67.0%)	316 (64.8%)	
Other	68 (39.3%)	104 (33.0%)	172 (35.2%)	
Sexual orientation (*n*; %)				**0.049**
Heterosexual	200 (82.0%)	180 (73.8%)	380 (77.9%)	
Homosexual	2 (1.2%)	7 (2.2%)	9 (1.8%)	
Bisexual	25 (14.5%)	48 (15.2%)	73 (15.0%)	
Asexual	2 (1.2%)	7 (2.2%)	9 (1.8%)	
Pansexual	5 (2.9%)	1 (0.3%)	6 (1.2%)	
Other	7 (4.0%)	4 (1.3%)	11 (2.3%)	
Marital status (*n*; %)				0.348
Single	119 (68.8%)	211 (67.0%)	330 (67.6%)	
In a relationship	47 (27.2%)	96 (30.5%)	143 (29.3%)	
Married	7 (4.0%)	6 (1.9%)	13 (2.7%)	
Separated	0 (0.0%)	2 (0.6%)	2 (0.4%)	
Eating disorder risk^*a*^(*n*; %)				0.120
Yes	39 (22.5%)	93 (29.5%)	132 (27.0%)	
No	134 (77.5%)	222 (70.5%)	356 (73.0%)	
Lifetime eating disorder diagnosis (*n*; %)				1.000
Yes	14 (8.1%)	26 (8.3%)	40 (8.2%)	
No	159 (91.9%)	289 (91.7%)	448 (91.8%)	

Note. Excluded participants were those removed due to low compliance. ^ chi-square test for categorical variables, *t*-tests for continuous variables. Significant *p* values bolded. *M* = mean, *SD* = standard deviation. ^a^ Based on a pre-defined cut-off score of ≥ 20 on the Eating Attitudes Test-26 (EAT-26; Garner et al., 1982)

### Measures

#### Baseline and trait-based measures

**Demographics**. Participants provided self-report information regarding age, weight and height (to calculate BMI; kg/m^2^), primary language, the highest level of education completed, ethnicity, marital status, sexual orientation, and lifetime eating disorder diagnosis.

**Self-objectification**. The Self-Objectification Questionnaire (SOQ; [27]) was used to assess participant’s trait-level self-objectification. Participants ranked 12 body attributes based on importance to their self-concept along a 12-point response scale from 1 (*least impact*) to 12 (*greatest impact*). Scores were computed by subtracting the sum of the six competence-based attributes (e.g., health, muscular strength etc.) from the sum of the six appearance-based attributes (e.g., sex appeal, physical attractiveness etc.). Scores ranged from − 36 to 36, with higher scores indicating greater self-objectification. Construct validity for this measure has been verified [27].

**Eating disorder risk**. Participants also completed the Eating Attitudes Test-26 (EAT-26; [26]), which assessed their attitudes and behaviours related to eating: 26 items were rated on a 6-point scale from 0 (*never*) to 5 (*always*). A total score ≥ 20 indicated a high level of eating disorder risk [26]. Internal consistency in the current study was strong: omega = 0.95.

#### State-based measures

**Social media-based appearance comparisons**. Participants’ engagement in appearance comparisons was measured by first asking “*Since the last survey*,* how much have you engaged in appearance comparisons?”* which was rated on an 11-point response scale from 0 (*not at all*) to 10 (*constantly*). Participants who made appearance comparisons (i.e., scored ≥ 1) were asked two additional questions regarding their most recent comparison: (1) “*In what context was this made?”* with response options of social media (e.g., Facebook, Instagram etc.), magazine, television/movie, in person, or other; and (ii) “*How did you think you looked compared to the other individual?*”, with responses rated on a 5-point response scale (*much worse*,* worse*,* the same*,* better*,* or much better*). For our study, analyses were restricted to social media-based comparisons. For the direction-of-comparison measure, responses of *worse* and *much worse* were coded as upward comparisons; responses of *same* were coded as lateral comparisons; and responses of *better* and *much better* were coded as downward comparisons. These items have been successfully utilised in previous EMA studies [11].

**Body satisfaction**. Body satisfaction was measured using the item *“How satisfied are you with your body right now?”*, rated on an 11-point response scale from 0 (*not at all*) to 10 (*completely satisfied*). This single-item approach has been shown to capture momentary fluctuations in body satisfaction in prior EMA studies [28, 29].

**Urges to engage in disordered eating**. DE urges were captured by asking participants whether they experienced an urge since the last survey to (1) *“consciously restrict food intake to control weight/shape”* (restrict food intake); (2) *“eat a large amount of food relative to what others would eat in the same situation/time”* (overeat); or (3) *“engage in at least 15 minutes of exercise to control weight/shape”* (exercise). Items were scored 1 (*yes*) and 0 (*no*). All current DE items have been previously utilised in EMA studies assessing DE urges [28–30].

### Procedure

The university’s ethics committee approved this study. Participants were provided with a web link to access the online survey platform, Qualtrics. After consenting, participants received a self-generated unique identifier and then completed the baseline survey collecting demographic information and trait-based (self-objectification, eating disorder risk) measures. Following completion, participants were emailed comprehensive instructions on how to use the custom-built EMA smartphone application, *SEMA3* [31]. *SEMA3* generated a unique code per participant which the researcher manually entered into Qualtrics to link the baseline and EMA data. Participants were asked to engage with the survey as much as possible and advised that their compliance was monitored; those with low compliance (< 50%) were emailed friendly reminders to complete more surveys.

Designed to commence the morning after completion of the baseline survey, *SEMA3* initiated push notifications at semi-random intervals, six times a day, between 9:00 AM and 10:00 PM, for seven days (maximum 42 assessments). At each signal, participants were asked to complete a 2-minute assessment of appearance comparison, body satisfaction, and DE urges. Random interval scheduling functioned to optimise the representativeness of the sample and reduce habituation, and the brief design was intended to minimise participant burden [32]. Upon completion of the entire study, REP-based participants received 2 units of course credit, whilst community-based participants entered a draw to win one of five $100 (AUD) vouchers.

### Data analytical plan

#### Data screening and preliminary analyses

Data pre-processing and analyses were performed in R version 4.0.3 [33]. The quality of the baseline and EMA-based data was examined before running the main analysis. As the baseline survey required complete responses (i.e., participants who failed to complete the baseline survey were removed from the sample), trait self-objectification data was not missing. There was no missing state-based (Level 1) within time points as participants either completed or did not start an EMA survey, and we did not impute for the EMA time points that were omitted. Although time points may have been missed due to a significant event (e.g., binge episode) or demographic trait (e.g., age) this cannot be ascertained directly from the data. To rectify this, we assessed reporting bias by correlating compliance (i.e., the proportion of EMA-based surveys completed out of the possible 42) with scores on demographic and trait (eating disorder risk) variables [34] and found no real basis for bias.

State-based outcome measures in the current hypotheses (social media-based appearance comparison, body satisfaction, DE urges) were examined for reactivity and time-related effects, on the presumption that these outcomes may vary depending on the time of the day, day of the week, and order of the assessment across the total 7-day period (i.e., Day 1, Day 2 etc.; [35]). Any significant time-related or reactivity effects were retained as covariates in the final models to account for any effects on the outcome measures. Order of assessment and/or time of day were identified as significant across different models. Results of these analyses may be obtained from the corresponding author upon reasonable request.

#### Hypothesis testing

Given the hierarchical nature of EMA data, multilevel modelling was deemed most suitable for our analysis [36]. Our main analyses (Hypotheses 1 and 2) focused on within-person effects to examine how momentary changes in our variables of interest related to subsequent changes in outcomes. Hypotheses regarding state-based associations [Hypotheses 1 (appearance comparison ◊ body satisfaction/DE urges) and 2 (body satisfaction/DE urges ◊ appearance comparisons)] involved regressing outcome variables onto predictor variables at the previous time point to enable the evaluation of prospective effects. In these models, predictor variables were group-mean-centred to disambiguate within-person effects. As the EMA items for appearance comparisons referred to behaviour since the *prior* assessment point, scores on body satisfaction and DE urges when modelled as predictors were lagged (*t* – 1) to ensure that predictor data reflected time preceding outcome data, which is consistent with prior EMA research [5]. The *lmer* function with a Gaussian distribution was used for continuous outcomes (body satisfaction), while the *glmer* function with a binomial distribution was used for binary outcomes (social media-based appearance comparisons; DE urges). Bi-directionality was inferred from two significant uni-directional relationships for the relevant variables.

In all models, comparisons (upward; downward; lateral) concerned those made via social media. To test Hypothesis 3, we examined cross-level interactions by including interaction terms with trait self-objectification (a between-person variable) in a subsequent step. This allowed us to investigate how a stable individual difference factor might moderate the within-person relationships explored in Hypotheses 1 and 2. Effect sizes for odds ratios (ORs) were interpreted according to Cohen’s [37] rule of thumb: OR = 1.68, 3.47, and 6.71 where equivalent to Cohen’s *d* = 0.2 (small), 0.5 (medium), and 0.8 (large), ORs less than 1 (indicating a negative association) were converted into numbers greater than 1 (positive association) to assist with comparability across ORs.

#### Power analyses

Using the *powerlmm* package [38] in R to estimate post hoc power for a multilevel model, the final sample of 315 participants was deemed sufficient to detect small effects (> 5% variance explained) with > 0.80 power (alpha = 0.05) under the following plausible assumptions: (1) intraclass correlations ranging 0.4–0.7; (2) average cluster size of 25–34 reflecting EMA compliance rates of ~ 60%–~80%; and (3) small variance in random slope for Level 1 effects. These effects are consistent with the results from EMA studies on body image (e.g., [28]).

## Results

### Preliminary analysis

#### Compliance

Participants in the final sample (*N* = 315) completed an average of 33.4 (*SD* = 5.85) out of 42 possible EMA surveys (78.8%) across the 7-day assessment period. Compliance rates were not significantly related to trait self-objectification (*r* = .00, *p* = .970), age (*r* = .05, *p* = .388), BMI (*r* = − .03, *p* = .604), primary language (*t* = -0.78, *p* = .437), ethnicity (*F* = 0.39, *p* = .817), educational attainment (*F* = 0.13, *p* = .941), sexual orientation (*F* = 1.06, *p* = .383), marital status (*F* = 2.01, *p* = .112), eating disorder risk (*t* = 1.44, *p* = .153), or lifetime eating disorder diagnosis (*t* = -0.32, *p* = .752). Before exclusions, the full sample (*N* = 488) completed an average of 28.3 (*SD* = 11.3) out of 42 surveys (67.4%). In this sample, only sexual orientation was significantly related to compliance (*F* = 2.72, *p* = .046). Full compliance analyses for all variables are available in the Supplementary Material.

#### Descriptive statistics

As displayed in Table 2, across the sample, at the state level, levels of body satisfaction were slightly below the scale’s midpoint. Regarding DE urges, those to restrict food intake were reported the most frequently, followed by urges to exercise, and lastly urges to overeat. Upward social media-based appearance comparisons were reported most frequently, followed by lateral comparisons which were recorded approximately a third of as much of the time. Downward social media-based appearance comparisons were reported the least frequently. At the trait level, the sample as a whole reported levels of self-objectification that were above the scale’s midpoint (i.e., suggesting a general tendency to self-objectify).

**Table 2:** Descriptive statistics for Level-1 and Level-2 variables

Variables	*M* ± *SD* / *n* (%)	Range
Level-1		
Body satisfaction	4.56 ± 1.75	0–10
Urge to overeat	420 (5%)	0–1
Urge to exercise	1028 (12%)	0–1
Urge to restrict food intake	1357 (16%)	0–1
Upward comparison	1671 (20%)	0–1
Lateral comparison	574 (7%)	0–1
Downward comparison	112 (1%)	0–1
Level-2		
Self-objectification	1.94 ± 18.85	-36–+36

Note. *M* = mean, *SD* = standard deviation. *n* = the number of instances in which the event occurred (across all EMA surveys completed) and then provides this as a proportion in brackets. Level-1 variables are state measures, whereas Level-2 variables are trait measures. All comparisons are social media-based appearance comparisons

Before removing participants who did not engage in social media-based appearance comparisons across the EMA phase, we also compared appearance comparisons across contexts (in-person, traditional media, social media, and ‘other’). The frequency of appearance comparisons on social media far exceeded any other context, but the direction of these comparisons (upward, lateral, downward) was comparable across contexts (data available upon request from the corresponding author).

### Multilevel models

#### ***Upward***,*** Lateral***,*** and downward appearance comparisons predicting body satisfaction and DE urges at the state level (H1)***

All effects reported in Tables 3 and 4 represent within-person associations. As shown in Table 3, engaging in upward social media-based appearance comparisons, relative to no comparison, predicted a subsequent decrease in state body satisfaction and an increase in urges to restrict food intake, exercise, and overeat. Additionally, engaging in lateral and downward comparisons, relative to no comparison, predicted a subsequent increase in state body satisfaction. Unexpectedly, lateral and downward comparisons, relative to no comparison, also predicted a subsequent increase in urges to exercise. Lateral and downward comparisons did not significantly predict changes in urges to restrict food intake or overeat. Effect sizes for most significant associations were small.

**Table 3:** Multilevel models testing within-person effects of appearance comparisons’ predicting body satisfaction and DE (hypothesis 1)

	State body satisfaction		Urge to restrict food intake		Urge to exercise		Urge to overeat	
Predictors	*b (95% CIs)*	*p*	*OR (95% CIs)*	*p*	*OR (95% CIs)*	*p*	*OR (95% CIs)*	*p*
None vs. upward^	-0.35 (-0.22, -0.48)	**< 0.001**	2.36 (1.90, 2.92)	**< 0.001**	2.71 (2.16, 3.40)	**< 0.001**	1.65 (1.22, 2.22)	**0.001**
None vs. lateral^	0.27 (0.05, 0.49)	**0.017**	1.38 (0.96, 1.98)	0.084	2.13 (1.47, 3.08)	**< 0.001**	1.08 (0.62, 1.87)	0.795
None vs. downward^	1.08 (0.66, 1.49)	**< 0.001**	1.31 (0.64, 2.68)	0.458	1.99 (1.05, 3.76)	**0.034**	1.23 (0.50, 3.00)	0.656

*Note. b* = unstandardized coefficient, OR = odds ratio, CI = confidence interval. Significant *p* values are bolded. ^No comparison is reference category in dummy coded appearance comparison direction variables

**Table 4:** Multilevel models testing within-person effects of body satisfaction and DE predicting appearance comparisons (hypothesis 2)

	None vs. upward		None vs. lateral		None vs. downward	
Predictors	*OR (95% CIs)*	*p*	*OR (95% CIs)*	*p*	*OR (95% CIs)*	*p*
State body satisfaction	-1.09 (-1.03, -1.16)	**0.002**	-1.10 (-0.99, -1.22)	0.087	-0.73 (-0.59, -0.91)	**0.005**
Urge to restrict food intake	1.35 (1.03, 1.78)	**0.031**	1.99 (1.16, 3.42)	**0.012**	4.87 (1.82, 13.00)	**0.002**
Urge to exercise	1.25 (0.93, 1.66)	0.134	1.86 (1.07, 3.20)	**0.027**	1.93 (0.69, 5.42)	0.213
Urge to overeat	1.15 (0.77, 1.71)	0.501	0.87 (0.34, 2.28)	0.785	2.84 (0.72, 11.20)	0.137

Note. OR = odds ratio, CI = confidence interval. Significant *p* values are bolded. ^No comparison is reference category in dummy coded appearance comparison direction variables

#### ***Body satisfaction and DE urges predicting upward***,*** lateral***,*** and downward appearance comparisons at the state level (H2)***

As displayed in Table 4, lower levels of state body satisfaction predicted an increased likelihood of engaging in upward and downward social media-based appearance comparison, relative to no comparison. Higher levels of urges to restrict food intake predicted an increased likelihood of engaging in all types of social media-based appearance comparison (upward, lateral, and downward), relative to no comparison. A stronger urge to exercise was associated with increased odds of lateral comparison versus no comparison. Effect sizes for these significant associations were negligible to small, except for the association between urge to restrict food intake and downward comparison, which was medium (OR = 4.87). No other predictor effects were significant.

#### Moderating effect of trait-based self-objectification (H3)

As seen in Tables 5 and 6 depicting cross-level interactions, trait self-objectification (between-person) significantly moderated only four of the possible 24 within-person state-based effects of appearance comparisons on body satisfaction and DE urges (H1) and vice versa (H2). Specifically, trait self-objectification moderated the effect of lateral comparisons (relative to no comparison) on state body satisfaction. Higher trait self-objectification was associated with a stronger positive relationship between lateral comparisons and state body satisfaction, though the effect size was negligible. Additionally, trait self-objectification moderated the effect of state body satisfaction on engagement in upward comparisons (relative to no comparison). For individuals with higher trait self-objectification, lower state body satisfaction was more strongly associated with an increased likelihood of engaging in upward comparisons, though the effect size was negligible. Furthermore, trait self-objectification moderated the effect of the urge to overeat on engagement in both upward and lateral comparisons (relative to no comparison). Higher trait self-objectification was associated with greater odds of engaging in upward and lateral comparisons when experiencing urges to overeat. No other moderation effects were significant, indicating that the relationship between appearance comparisons and body satisfaction/DE urges, as well as the reverse relationships, were mostly consistent across levels of trait self-objectification.

**Table 5:** Cross-level interactions: trait self-objectification (between-person) moderating within-person effects of appearance comparisons’ on body satisfaction and DE (hypothesis 3a)

	State body satisfaction		Urge to restrict food intake		Urge to exercise		Urge to overeat	
Predictors	*b (95% CIs)*	*p*	*OR (95% CIs)*	*p*	*OR (95% CIs)*	*p*	*OR (95% CIs)*	*p*
None vs. upward*SO	-0.00 (-0.01, 0.01)	0.763	1.00 (0.99, 1.01)	0.970	0.99 (0.98, 1.01)	0.223	1.00 (0.98, 1.01)	0.766
None vs. lateral*SO	0.01 (0.00, 0.03)	**0.034**	1.01 (0.98, 1.03)	0.657	1.00 (0.98, 1.03)	0.686	1.03 (0.99, 1.06)	0.112
None vs. downward*SO	-0.00 (-0.03, 0.03)	0.954	1.03 (0.95, 1.11)	0.476	1.03 (0.94, 1.12)	0.587	1.03 (0.97, 1.09)	0.305

*Note. b* = unstandardized coefficient, OR = odds ratio, CI = confidence interval, SO = trait-based self-objectification. Significant *p* values are bolded. ^No comparison is reference category in dummy coded appearance comparison direction variables

**Table 6:** Cross-level interactions: trait self-objectification (between-person) moderating within-person effects of body satisfaction and DE on appearance comparisons (hypothesis 3b)

	None vs. upward		None vs. lateral		None vs. downward	
Predictors	*OR (95% CIs)*	*p*	*OR (95% CIs)*	*p*	*OR (95% CIs)*	*p*
State body satisfaction*SO	-1.00 (-1.00, -0.99)	**0.044**	-1.00 (-1.00, -0.99)	0.692	-0.99 (-1.00, -0.98)	0.126
Urge to restrict food intake*SO	1.01 (0.99, 1.02)	0.295	1.00 (0.97, 1.02)	0.880	1.02 (0.97, 1.07)	0.454
Urge to exercise*SO	1.01 (1.00, 1.03)	0.077	0.98 (0.95, 1.01)	0.259	1.04 (0.98, 1.10)	0.180
Urge to overeat*SO	1.00 (1.00, 1.01)	**< 0.001**	1.05 (1.05, 1.05)	**< 0.001**	0.96 (0.89, 1.03)	0.268

Note. OR = odds ratio, CI = confidence interval, SO = trait-based self-objectification. Significant *p* values are bolded. ^No comparison is reference category in dummy coded appearance comparison direction variables

## Discussion

The present study was the first to use EMA to examine the bi-directional relationships between social media-based appearance comparison (upward, lateral, and downward) with body satisfaction and DE urges throughout women’s daily lives, as moderated by self-objectification [20, 21]. We hypothesised that (1) upward comparisons would predict lower levels of body satisfaction and higher levels of DE urges, while lateral and downward comparisons would predict higher body satisfaction and lower DE urges; (2) lower body satisfaction and higher DE urges would predict upward comparisons, while higher body satisfaction and lower DE urges would predict lateral or downward comparisons; and (3) self-objectification would moderate these relationships, strengthening the effects. Consistent with expectations, we observed significant bi-directional relationships in the expected directions between upward comparisons and body satisfaction (negative) and urges to restrict food intake (positive). Unexpectedly, greater urges to restrict food intake predicted an increased likelihood of all types of comparisons, not just upward comparisons. Additionally, we found a significant bi-directional relationship between state body satisfaction and downward comparisons, though in partially unexpected directions. Contrary to expectations, we also found a significant bi-directional relationship between lateral comparisons and urges to exercise in an unexpected positive direction. We observed a uni-directional relationship between upward comparisons and urges to overeat. Most observed effects were small. Self-objectification only moderated four of the possible relationships, with negligible effects.

### Bi-directional effects of social media-based appearance comparisons with body satisfaction and DE urges at the state level

Consistent with expectations, our findings revealed significant micro-longitudinal bi-directional relationships between upward social media-based appearance comparisons and both body satisfaction and urges to restrict food intake. Engaging in upward (relative to no) comparisons predicted lower body satisfaction and increased restrictive urges, while these states in turn predicted increased engagement in upward comparisons. These cyclical within-person effects broadly align with previous EMA research observing unidirectional effects between these constructs [5, 11] and support the circle of objectification framework [20].

Theoretically, for example, engaging in upward appearance comparisons on social media may reinforce a perceived discrepancy between oneself and sociocultural appearance ideals, increasing the desire to restrict food intake and control body weight/shape. This, in turn, may prompt continued comparison with the idealised standard, creating an unhealthy circular process.

Unexpectedly, urges to restrict food intake predicted an increased likelihood of all comparison types, not just upward, suggesting a more complex relationship between DE urges and appearance comparisons than initially hypothesized. Theoretically, individuals experiencing urges to restrict may engage in various types of comparisons as part of a broader pattern of appearance-focused behaviour [39, 40], regardless of the direction of comparison.

Additionally and unexpectedly, we observed a bi-directional positive relationship between lateral comparisons (relative to no comparison) and urges to exercise. Theoretically, lateral comparisons may motivate exercise as a means of maintaining or slightly improving one’s perceived status relative to peers. Conversely, higher exercise urges may prompt lateral comparisons to gauge one’s progress.

Lastly, contrary to expectations, we found a negative bi-directional relationship between state body satisfaction and downward comparisons (relative to no comparisons). Engaging in downward comparisons predicted higher body satisfaction, whilst higher body satisfaction predicted a lower likelihood of engaging in downward comparisons. These findings suggest a more complex relationship than initially hypothesized. While downward comparisons may boost state body satisfaction in the short term, when individuals experience higher momentary body satisfaction, they may not feel the immediate need to compare themselves to others they perceive as less attractive. Collectively, current findings challenge any assumptions that all appearance comparisons are uniformly and/or necessarily detrimental and highlight the potential for some types of comparisons to have positive effects on body image. Present findings also highlight the fluctuating nature of body satisfaction/DE urges and comparison behaviours within individuals over time, emphasizing the importance of considering these constructs at the state level rather than as stable traits.

Partially consistent with expectations, uni-directional relationships were found between upward appearance comparisons and urges to overeat, as well as between lateral comparisons and urges to exercise. Within persons, engaging in upward (relative to no) appearance comparisons predicted increased urges for overeating at the subsequent assessment point, aligning with previous research linking upward comparisons to DE behaviours [5, 11]. However, these urges did not predict subsequent upward comparisons. Additionally and unexpectedly, lateral comparisons predicted increased urges to exercise, but not vice versa. These findings challenge our initial assumption about the directionality of these relationships and highlight the need for a more nuanced understanding of how different types of comparisons relate to specific eating and exercise behaviours.

The observed patterns may be influenced by personality traits. The unique predictive relationship between restrictive urges and all comparison types may be partially explained by trait-level harm avoidance, which has been linked to restrictive eating behaviours [41]. Individuals high in harm avoidance may engage in various types of comparisons as a strategy to minimize perceived risks related to body image and weight; e.g., curating one’s social media feed in a way that keeps inspiration/strategies to reach the desired appearance goal nearby, such as seeking workout/meal plans from fitness influencers, paradoxically reinforcing negative body-related schemas. Conversely, the absence of predictive relationships between overeating urges and comparisons might relate to trait level impulsivity which has been associated with binge eating [42], potentially leading to less deliberate comparative behaviour. Future research should investigate the possible influence of these personality traits to better elucidate the complex interplay between social media use, body image, and eating behaviours, potentially informing targeted interventions.

### Moderating effects of self-objectification

Contrary to predictions, trait-level self-objectification only moderated four out of a possible 24 state-level relationships; all were uni-directional. In other words, self-objectification was a significant moderator when the predictor and outcome were modelled in *one* direction (i.e., the predictor and outcome were positively *or* negatively related). Although current findings provided limited support for our proposed moderation model, four moderation effects were observed. For women with higher trait-level self-objectification, lateral (relative to no) appearance comparisons predicted increased body satisfaction and urges to overeat; whilst reduced body satisfaction predicted increased engagement in upward (relative to no) comparisons. The latter finding is broadly consistent with research supporting the moderating effect of self-objectification on the indirect relationship between selfie-viewing and BD via appearance comparisons [24]. The findings regarding DE were novel as prior researchers have not assessed the moderating effect of self-objectification on the relationship between appearance comparisons and eating concerns.

Drawing from notions by Fuller-Tyszkiewicz [5], the unexpected and small number of moderating effects for trait self-objectification observed in our study may suggest that self-objectification (and other dispositional traits associated with elevated appearance investment) make certain appearance-related behaviours (e.g., upward appearance comparisons) more common—with subsequent impact on body satisfaction and DE—rather than making the effects of these behaviours larger. Nonetheless, current conclusions are tentative pending replication. Future studies should assess whether the current model (i.e., moderation) or an alternative model (i.e., mediation) better explains the relationships.

### Limitations, strengths, and future directions

several limitations that should be acknowledged. First, our findings were derived from a predominately undergraduate cohort of heterosexual women of Caucasian or Asian ethnicity. Future research must evaluate generalisability to other important populations such as men [43], other sexual orientations [44], and other ethnicities [45] that may experience heightened vulnerability to eating disorders. For instance, sexual minorities experience greater DE behaviours than their heterosexual counterparts [46]. Future research must also ultimately test and verify these relationships in a clinical eating disorder sample.

Second, our state-based DE items may not have adequately captured DE, and thus, effects should be interpreted with caution. For instance, we did not clearly define the degree of food restriction and the *‘urge to engage in at least 15 min of exercise to control weight/shape’* may not have accurately captured excessive/dysregulated exercise. Additionally, our overeating variable did not meet the Diagnostic and Statistical Manual of Mental Disorders (DSM-5) criterion of a binge eating episode (e.g. eating a larger amount in a discrete period [i.e., 2-hour period] and losing control over eating; [47]). Future EMA studies should define the degree of restriction and measure the attitudes and affect (e.g., guilt) motivating the exercise urge for more nuanced and intentional research into the effects. Future EMA studies should also capture binge eating according to the DSM-5 [47] criterion, and assess purging as binge eating/purging offers the closest representation of bulimic symptomatology.

Third, our broad, single-item measure of social media-based appearance comparisons failed to capture other aspects of interest, such as; specific comparison targets (e.g., friend, close peer, acquaintance, celebrity/model), the perceived attainability of different targets; and differences across platforms which are more visual/appearance-based (e.g., Instagram, Facebook) versus those more text-based (e.g., Twitter; [48]). Moreover, participants reported on their most recent comparison, however, it is plausible that participants compared themselves to multiple targets since the last survey, given the amount of visual information on social media. Future EMA researchers should consider incorporating more detailed assessment items to assess the influence of these factors.

Fourth, future research should consider including all appearance comparison contexts (e.g., in-person, traditional media, social media) and examining context as a potential moderator. This could provide insights into whether comparison effects differ across contexts, further refining our understanding of these relationships.

Finally, our data were collected throughout the coronavirus disease 2019 (COVID-19) pandemic, and thus, might not generalise to other contexts. Indeed, the frequency of appearance comparisons on social media far exceeded those in other contexts, but the direction of these comparisons (upward, lateral, downward) was comparable across contexts.

Notwithstanding the aforementioned limitations, our study had notable strengths. First, in a field dominated by experimental methodology (e.g., [12, 49]) we employed EMA which provided naturalistic, micro-longitudinal data. This enabled the novel assessment of temporal bi-directional patterns and momentary processes, whilst increasing ecological validity and the generalisability of findings [50]. Second, our study had a much larger sample size than other EMA studies in the field with a similarly demanding protocol (e.g., [11]), which increased the statistical power to undertake meaningful analyses.

## Conclusions

This study reveals complex, bi-directional relationships between social media-based appearance comparisons, body satisfaction, and DE urges in women’s daily lives. Our findings challenge simplistic views of appearance comparisons, highlighting that their effects can vary based on comparison direction and specific outcomes. Notably, upward comparisons were linked to lower body satisfaction and increased restrictive urges, with these states predicting further upward comparisons, perpetuating a potentially harmful cycle. Unexpectedly, restrictive urges predicted all comparison types, suggesting a pervasive influence of DE tendencies on social media behaviour. Also unexpectedly, lateral comparisons were associated with increased exercise urges, with these states predicting further lateral comparisons. These unexpected findings may reflect underlying cognitive biases and coping strategies observed in eating disorder populations, at least in the short term, and suggest that future ED research and possibly interventions should consider the varied roles of downward and lateral comparisons. Moreover, the reciprocal nature of these relationships highlights the potential importance of targeting both comparison behaviours and body image/eating concerns simultaneously in prevention and/or intervention strategies. Our findings provide limited support for the moderating effect of trait self-objectification. Future studies should explore these dynamics across diverse populations, employ more refined measures over extended periods, and examine the influence of various personality traits to increase understanding and ultimately inform targeted strategies to improve body image and eating concerns in the context of social media use.

### Electronic supplementary material

Below is the link to the electronic supplementary material.

Supplementary Material 1

## Data Availability

The data is available upon request.
