# Supplementary material for: Daily Bi-directional effects of women’s social media-based appearance comparisons, body satisfaction, and disordered eating urges
Source: J Eat Disord. 2024 Sep 3;12:129. doi: 10.1186/s40337-024-01096-8 (PMC11369994; doi:10.1186/s40337-024-01096-8)
Supplement: Supplementary file 1 — Supplementary Material 1 [file 40337_2024_1096_MOESM1_ESM.docx]

**Supplementary Material**

Compliance statistics across full sample (*N* = 488):

*M* = 28.3 (*SD* = 11.3)

Median (min, max) = 32.0 (1.0, 45.0)

Relationship between compliance rates and trait/demographic variables across full sample:

- Age (r = 0.01, p = .792)
- BMI (r = 0.04, p = .379)
- Primary language (*t* = -0.33, *p* = .744)
- Ethnicity (*F* = 0.77, *p* = .549)
- Educational attainment (*F* = 1.78, *p* = .149)
- Sexual orientation (*F* = 2.72, *p* = .046)
- Marital status (*F* = 0.21, *p* = .888)
- Eating disorder risk (*t* = 1.17, *p* = .244)
- Trait self-objectification (r = 0.02, p = .603)
